# Clinical Management of Severe *Cupriavidus gilardii* Superinfection After Influenza a Virus Pneumonia: A Case Report and Literature Review

**DOI:** 10.3390/idr18020024

**Published:** 2026-03-13

**Authors:** Chenxia Guo, Cuihong Sun, Jiajia Zheng, Qingtao Zhou, Ying Liang

**Affiliations:** 1 Department of Respiratory and Critical Care Medicine, Peking University Third Hospital, Beijing 100191, China; guochenxia1990@163.com (C.G.); qtzhou75@163.com (Q.Z.); 2 Department of Respiratory and Critical Care Medicine, Peking University Third Hospital Qinhuangdao Hospital, Qinhuangdao 066000, China; 17349848184@163.com; 3 Laboratory Department, Peking University Third Hospital, Beijing 100191, China; zhengjiajia@bjmu.edu.cn

**Keywords:** *Cupriavidus gilardii*, bloodstream infection, septic shock, multiple organ failure

## Abstract

Background: *Cupriavidus* is an aerobic Gram-negative bacterium and a rare conditional pathogen that mainly infects immunocompromised patients or those undergoing invasive procedures. Methods: We present the case of a 70-year-old male with diabetes mellitus who developed septic shock following influenza A virus (IAV) pneumonia. *Cupriavidus gilardii* (*C. gilardii*) was identified in his blood and sputum samples. Through a literature review, we identified 31 reported cases of *Cupriavidus* infections. Clinical data, including demographic information, clinical characteristics, comorbidities, laboratory results, *Cupriavidus species*, treatment, and clinical outcomes, were collected. Results: Among these 32 patients (including our patient), 23 were male (71.9%) and 9 were female (28.1%). The median patient age was 32.5 (2.12–70) years. Most patients had relevant risk factors or comorbidities before *Cupriavidus* infection, including exposure to polluted environments and recent invasive procedures (68.9%). Among these cases, *Cupriavidus pauculus* was the most common strain, accounting for 56.3% of cases. The mortality rate was the highest for *Cupriavidus pauculus* infections. Conclusions: *Cupriavidus* is a rare opportunistic pathogen in patients with compromised immune function. Early identification of pathogen and timely treatment are crucial. When traditional microbiological detection methods encounter difficulties, gene sequencing can be used as an auxiliary diagnostic tool and can further predict drug resistance. Targeted anti-infection treatment is effective in most cases, but some severe infection cases may lead to death due to serious complications.

## 1. Introduction

*Cupriavidus* is a genus of conditionally pathogenic nonfermenting, aerobic Gram-negative bacillus with flagellar morphology that was first identified by Makkar and Casida in 1987 and belongs to the Burkholderia family of the phylum β-Proteobacteria. Common strains include *Cupriavidus gilardii* (*C. gilardii*), *Cupriavidus pauculus* (*C. pauculus*), *Cupriavidus respiraculi* (*C. respiraculi*), *Cupriavidus metallidurans* (*C. metallidurans*), and *Cupriavidus taiwanensis* (*C. taiwanensis*) [1]. The morphology of the colonies on the blood agar plate was similar to that of *Pseudomonas aeruginosa*, with round, moist, milky yellow colonies with neat edges. The strain is catalase-positive and oxidase-negative, with optimal growth at 37–40 °C, in the presence of 0.5% NaCl and at pH 7.0 [2].

*Cupriavidus* is ubiquitously present in the environment and rarely causes human infections. We reported a severe case of *C. gilardii* infection following IAV pneumonia, resulting in death due to multi-organ failure. A literature search identified a total of 31 case reports of *Cupriavidus* infections, of which 22 cases improved with targeted antimicrobial therapy, and 9 cases died from severe infection complications.

### Case Presentation

On 21 November 2023, a 71-year-old male was admitted to our hospital with a one-month history of cough and expectoration and 4 days of dyspnea. One month prior to admission, he developed an irregular fever with a maximum temperature of 38.5 °C, accompanied by cough and expectoration. Positive influenza A virus (IAV) nucleic acid test was detected, and a chest computed tomography (CT) scan revealed patchy infiltrates in both lungs. He was treated with oxygen therapy, oseltamivir for antiviral therapy, and piperacillin/tazobactam (4.5 g every 8 h) and levofloxacin (0.5 g once daily) for potential bacterial infection. Despite being discharged, he still experienced intermittent cough and expectoration. Four days before admission, his symptoms worsened, accompanied by progressive dyspnea. His routine blood test revealed a white blood cell count of 10.5 × 10^9^/L, a neutrophil count of 8.93 × 10^9^/L, and a C-reactive protein concentration of 165 mg/L. Arterial blood gas analysis (without oxygen supplementation) revealed a pH of 7.37, a PO_2_ of 46 mmHg, a PCO_2_ of 33 mmHg, and a lactate concentration of 0.8 mmol/L. Afterward, the patient was transferred to the emergency department of our hospital.

The medical history of this patient included hypertension, diabetes mellitus, and old cerebral infarction for several years.

Physical examination revealed a body temperature of 36.5 °C, pulse rate of 73 beats/min, respiratory rate of 24 breaths/min, and blood pressure of 116/65 mmHg. On admission, the patient was conscious, with auscultation revealing a few moist rales in both lungs and no pleural friction rub. Examination of the heart and abdomen revealed no significant abnormalities, and there was no edema in the lower extremities.

Laboratory evaluation at admission revealed a white blood cell count of 14.41 × 10^9^/L, a neutrophil percentage of 90%, a procalcitonin (PCT) concentration of <0.1 ng/mL, a ferritin concentration of 1341 ng/mL, and a serum IL-6 concentration of 18 pg/mL. The CD4^+^ T-cell count was within the normal range. Nucleic acid tests for influenza virus, SARS-CoV-2, respiratory syncytial virus and other respiratory pathogens were all negative (samples were collected via nasopharyngeal swabs). Sputum acid-fast staining was negative. His antinuclear antibody and antineutrophil cytoplasmic antibody results were negative.

**Figure 1:**
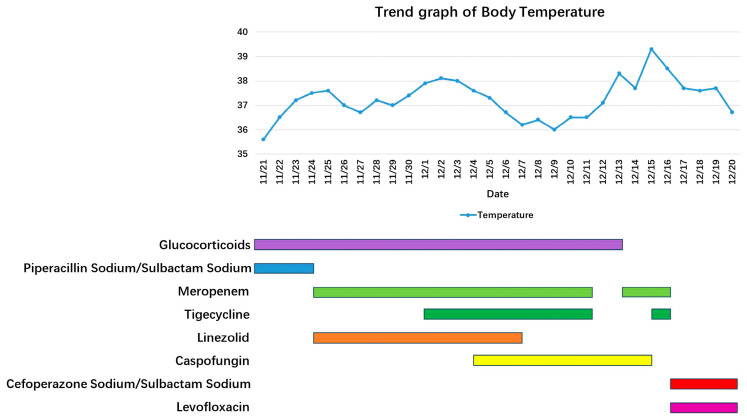
Timeline of treatment. The colored bars represent the duration of administration for each antimicrobial agent. Piperacillin/Sulbactam (**blue**), Meropenem (**green**), Tigecycline (**dark green**), Linezolid (**orange**), Caspofungin (**yellow**), Cefoperazone/Sulbactam (**red**), and Levofloxacin (**magenta**) were used at different stages to manage the patient’s condition.

**Figure 3:**
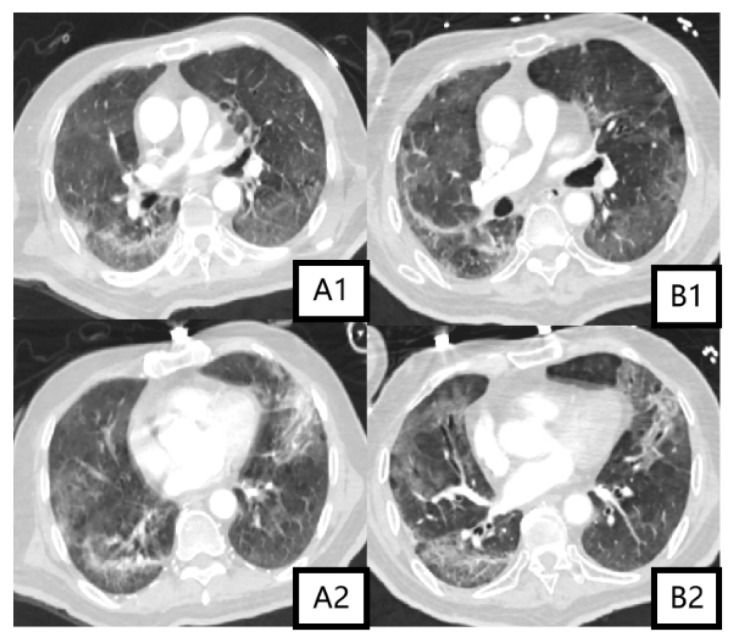
On the 10th hospital day, chest CT revealed multiple patchy ground-glass opacities in both lungs, with some irregular consolidation (30 November 2023; (**A1**,**A2**)). On the 17th day after admission, chest CT showed that ground-glass opacities and consolidation were partially absorbed in both lungs (7 December 2023; (**B1**,**B2**)).

The timeline of his treatment is illustrated in Figure 1, and changes in inflammatory indicators, the oxygenation index, and etiology are detailed in Table 1. On admission, a chest X-ray showed multiple patchy high-density shadows in both lungs (Figure 2①), with an oxygenation index of <250 mmHg (with a face mask at 5 L/min). Severe pneumonia was diagnosed, and the patient was given prone ventilation and empirical anti-infection treatment with 5 g of piperacillin-sulbactam every 8 h and 40 mg of methylprednisolone once daily. Bedside bronchoscopy and metagenomic next-generation sequencing (mNGS) of the bronchoalveolar lavage fluid (BALF) detected respiratory syncytial virus type A (RSV-A; sequence count 32) and herpes simplex virus type 1 (HSV-1; sequence count 11). On the fourth day of hospitalization, owing to further worsening of dyspnea and failure of noninvasive ventilation, the patient was intubated and given invasive mechanical ventilation. A follow-up chest X-ray showed progression of pulmonary infiltration lesions (Figure 2②). The treatment was adjusted to meropenem (1 g every 8 h) in combination with linezolid (0.6 g every 12 h). One week later, the patient’s condition improved. A repeat bronchoscopy was performed, and mNGS of the BALF revealed only HSV-1 (sequence count 17), while RSV-A was not detected. Chest imaging revealed partial absorption of the pulmonary inflammation compared with the previous findings (Figure 2③ and Figure 3(A1,A2)). The endotracheal tube was removed. However, after extubation, the patient developed fever again, with a white blood cell count of 17 × 10^9^/L. The (1,3)-*β*-D-glucan test and galactomannan test were positive, and subsequently, the patient developed septic shock. Caspofungin was administered in combination with tigecycline to target possible secondary fungal infections and drug-resistant Gram-negative organisms. The body temperature gradually returned to normal, while follow-up chest CT showed no significant changes in the bilateral lung lesions (Figure 3(B1,B2)). On the 24th day after admission, the patient developed fever again, with a chest X-ray showing worsening pulmonary exudation (Figure 2④). Blood cultures flagged positive for Gram-negative bacilli (Supplementary Explanation: Bacterial identification and antimicrobial susceptibility testing were performed with the VITEK-2 Compact system (bioMérieux, Lyon, France). The specimens were subjected to three-zone streaking on blood agar and China Blue agar, followed by overnight incubation at 37 °C in a 5% CO_2_ atmosphere. After incubation, single bacterial colonies were isolated for bacterial identification. Afterward, the patient was treated with 0.5 g of meropenem every 12 h in combination with 50 mg of tigecycline every 12 h. However, the patient still had fever, and rhabdomyolysis occurred. Blood culture results revealed *C. gilardii*. According to the antimicrobial susceptibility results, this isolate was resistant to meropenem and imipenem (both MIC values > 32 µg/mL) but susceptible to ceftriaxone (MIC value of 1.5 µg/mL) and levofloxacin (MIC value of 0.094 µg/mL). The antibiotic regimen was changed to 3 g of cefoperazone-sulbactam every 8 h in combination with 0.5 g of levofloxacin once daily. On the 29th day after admission, *C. gilardii* was also identified in his sputum culture, with the same antimicrobial susceptibility results as those from the blood culture (Table 2). Owing to further deterioration of renal function, the patient underwent bedside hemofiltration treatment. On the 30th day after admission, the patient’s respiratory failure worsened, and invasive mechanical ventilation had to be performed again. A Chest X-ray showed progression of bilateral lung lesions (Figure 2⑤). The patient’s condition deteriorated rapidly, and he ultimately died from septic shock and multiple organ failure.

## 2. Materials and Methods

### 2.1. Data Collection

**Figure 4:**
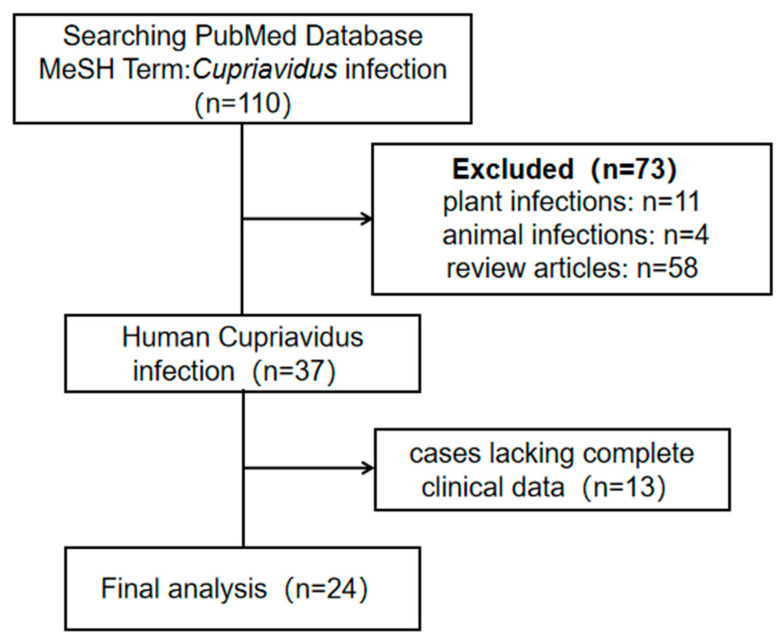
PRISMA flowchart.

We conducted a comprehensive search of the PubMed database from 1 January 2009 to 1 April 2025, using the MeSH term “*Cupriavidus* infection” without language restrictions. The search was limited to case reports and case series with complete clinical data. The inclusion criteria were as follows: (1) case reports or case series and (2) *Cupriavidus* infection confirmed by etiological or pathological examination. The exclusion criteria included the following: (1) plant infections; (2) animal studies or review articles; and (3) cases lacking complete clinical data. Articles were screened, and cases with *Cupriavidus* infection confirmed by etiological or pathological examination were included in our study (Figure 4). Clinical data, including demographic information, clinical characteristics, comorbidities, laboratory results, *Cupriavidus species*, treatment, and clinical outcomes, were collected.

### 2.2. Statistical Analysis

Continuous variables were presented as medians (interquartile ranges), while categorical variables were presented as numbers and percentages. SPSS 22.0 software (IBM Corp., Armonk, NY, USA) was used for statistical analysis.

## 3. Results

Initially, a total of 110 articles were retrieved through the search process. Of these, 24 met the inclusion criteria, collectively reporting 31 documented cases of *Cupriavidus* infection (Table 3). Among these 32 patients (including our patient), 23 were male (71.9%), and 9 were female (28.1%). The median age was 32.5 (2.12–70) years, ranging from 6 days after birth to 90 years (Table 4). Most patients had relevant risk factors or comorbidities before *Cupriavidus* infection, including exposure to polluted environments and recent invasive procedures (68.9%). Other risk factors included congenital diseases, hematologic disorders, solid tumors, the use of hormones and immunosuppressive agents, and autoimmune diseases. The routes of infection were predominantly hematogenous (75%) and respiratory (21.9%), with pathogens identified through blood and peripheral tissue cultures, as well as respiratory secretion cultures. Clinical manifestations included fever (71.9%), respiratory symptoms (cough, expectoration, and dyspnea) (31.3%), gastrointestinal symptoms (abdominal pain, vomiting, diarrhea, hematochezia, and jaundice) (15.6%), neurological symptoms (headache, altered consciousness, epilepsy, and opisthotonos) (12.5%), and skin or peripheral tissue infection manifestations (localized limb abscesses with pain and wound suppuration) (15.6%).

Among these cases, *C. pauculus* was the most common strain, accounting for 56.3% of cases, followed by *C. gilardii* (25%), *C. metallidurans* (15.6%), and *C. respiraculi* (3.1%). With respect to clinical outcomes, 22 patients improved with treatment, while 10 patients died (including our patient). The mortality rate was the highest for *C. pauculus* infections.

## 4. Discussion

*Cupriavidus* is widely distributed in nature and is commonly found in soil, plant roots, and particularly in water environments, including water supply systems. As a microorganism with low virulence, *Cupriavidus* rarely causes infections in humans. Opportunistic infections primarily occur in immunocompromised hosts or those with complex comorbidities, whereas infections in immunocompetent individuals are predominantly associated with invasive medical procedures. As of 2025, a total of 31 confirmed *Cupriavidus* infection cases have been reported (Table 3), with the earliest case dating back to 2009. The age of onset ranged from newborns to 90 years. Many patients had risk factors such as viral infections, the use of glucocorticoids or immunosuppressive agents, hematologic diseases, and catheter insertions. The sources of pathogens included 7 cases detected in sputum and bronchoalveolar lavage fluid, 24 cases in blood and catheter cultures, one case in bone marrow cultures, one case in cerebrospinal fluid cultures, four cases in skin abscesses and secretion cultures, and one case in fecal tests. Among them, 22 patients (68.7%) were discharged after targeted anti-infection treatment, while 10 (31.3%) died.

This study details a unique case involving a patient recovering from IAV pneumonia. Initial routine nucleic acid testing for respiratory pathogens was negative, while the nucleic acid sequence of RSV-A was subsequently detected by mNGS. However, subsequent conventional culture during the disease course revealed a secondary infection with a rare bacterium, revealing the dynamic evolution of pathogens during the infection process. In this case, mNGS served as a navigational tool, rapidly excluding common pathogens, whereas conventional culture played a key role in detecting the secondary bacterial infections. This highlights the critical role of a complementary diagnostic strategy combining precision medicine with traditional microbiology in the management of critical illness. The absence of the bacterium in the initial mNGS report suggested that secondary infections with rare bacteria can be insidious during the “immune window” following viral infection. The isolation of *C. gilardii* from both blood and bronchoscopic aspirate cultures, identified as a carbapenem-resistant strain, along with its complicated clinical course and fatal outcome, provides profound practical insights for managing such rare infections.

Although RSV-A nucleic acid sequences were detected in the first mNGS analysis, nasopharyngeal swab viral nucleic acid testing was negative, and no RSV-A signal was confirmed in the second mNGS assay. Accordingly, we conclude that RSV-A was not the primary causative pathogen in this patient. In contrast, IAV infection was definite, as the initial IAV nucleic acid test was positive. In clinical practice, the diagnosis of viral infection is primarily based on targeted nucleic acid positivity rather than mNGS findings. Although HSV-1 was detected, it was considered a colonizer due to the rarity of such infection, inconsistency with clinical and radiological findings, and the extremely low read count. Therefore, no other secondary infections were confirmed to have contributed to the pneumonia.

Analysis of the patient’s risk factors revealed that despite a normal CD4^+^ T-cell count on admission, he had received systemic corticosteroid treatment for acute lung injury and had a diabetes history. Moreover, he had undergone invasive procedures such as endotracheal intubation, urinary catheterization, and central venous catheter placement, all of which are high-risk factors for opportunistic infection. IAV infection is typically followed by secondary bacterial and fungal infections in the lungs [27,28]. A major complication of viral infection is bacterial colonization of the affected organ, which is associated with high morbidity and mortality. We propose the following mechanisms for this process: (1) Inflammatory response: Viral-induced inflammation disrupts the airway epithelial barrier and impairs mucociliary clearance, exacerbating airway obstruction and creating a susceptible environment for secondary infection. (2) Immune modulation: Viral infection compromises the host immune system, particularly the barrier function of the respiratory mucosa and local immune response, creating an “immune window” for opportunistic pathogen invasion and colonization. (3) Microbiota dysbiosis: A history of severe IAV pneumonia, disrupted the respiratory microbiota balance. This imbalance increases bacterial adhesion and invasion. As an opportunistic pathogen, *C. gilardii* typically colonizes immunocompromised or damaged tissues; the disrupted local environment likely facilitates its transition from colonization to invasive infection.

There is limited evidence regarding the antimicrobial susceptibility, optimal treatment, and duration (especially in severe cases) of *Cupriavidus* infections. Previous literature indicates that the majority of strains are susceptible to ciprofloxacin, while resistance to cefepime, cefotaxime, trimethoprim-sulfamethoxazole, piperacillin-tazobactam, and levofloxacin is rare [3].

The bacterial species within the genus *Cupriavidus* possess a variety of antibiotic resistance genes, and the presence of efflux pump genes such as CeoAB-OpcM, MexAB-OprM [29] and the MCR-5 gene may compromise efficacy [14,30]. According to previous case reports, *Cupriavidus* is a rare potential pathogen of ventilator-associated pneumonia (VAP), especially in immunocompromised populations [20,31]. Third-generation cephalosporins and ciprofloxacin have demonstrated significant efficacy in treating *Cupriavidus*-induced VAP and bacteremia [7]. For treatment of catheter-related bloodstream infections, it has been reported in the literature that if persistent colonization is suspected, catheter removal may be necessary. For patients for whom catheter removal is difficult, catheter lock therapy in combination with systemic antibiotics may be a safe option [5,6]. Although *Cupriavidus* can cause severe infectious diseases, most reports indicate that treatment with antibiotics is effective. Throughout the disease course, the patient experienced recurrent fever accompanied by leukocytosis and elevated inflammatory markers. Carbapenem-resistant *C. gilardii* was eventually isolated from both blood and sputum cultures. Despite adjusting the antimicrobial regimen based on susceptibility results and a subsequent downward trend in body temperature, the patient’s condition deteriorated rapidly. Ultimately, the patient succumbed to septic shock and multiple organ failure. However, these findings align with previous observations that mortality in *Cupriavidus* infections is typically not directly attributable to the pathogen itself but rather to associated underlying comorbidities or complications [5].

## 5. Conclusions

In summary, *Cupriavidus* is a rare opportunistic pathogen in patients with compromised immune function. Early identification of pathogen and timely treatment are crucial. When traditional microbiological detection methods encounter difficulties, gene sequencing can be used as an auxiliary diagnostic tool and can further predict drug resistance. Targeted anti-infection treatment is effective in most cases, but some severe infection cases may lead to death due to serious complications.

## Figures and Tables

**Figure 2:**
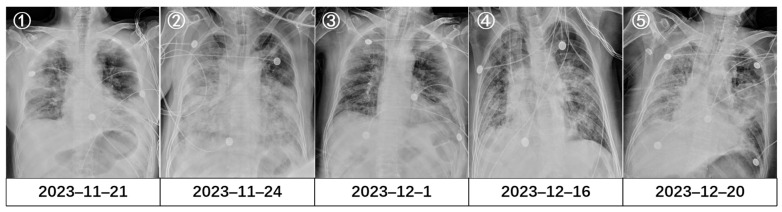
Changes in the Chest X-ray. **①** Chest X-ray on 21 November 2023 showed multiple patchy high-density shadows in both lungs. **②** Chest X-ray on 24 November 2023 showed progressive worsening of the pulmonary infiltrates and in-creased opacity. **③** Chest X-ray on 1 December 2023 revealed partial absorption of the pulmonary in-flammation compared with the previous findings. **④** Chest X-ray on 16 December 2023 showed wors-ening pulmonary exudation. **⑤** Chest X-ray on 20 December 2023 showed progression of bilateral lung lesions.

**Table 1:** Changes in inflammatory indicators, the oxygenation index and etiology.

Date	11–21	11–22	11–24	11–29	12–1	12–4	12–11	12–16	12–19	12–20
WBC	14	18	16	13	17	34	18	11	6.2	5.8
PCT	<0.1	-	0.13	<0.1	<0.1	0.28	0.11	0.39	0.83	0.65
PLT	187	203	133	99	142	205	110	89	29	17
PO_2_/FiO_2_	124		82	231	269	290	268	146	145	56
Respiratory Support	Face mask		NIV→IMV		Nasal cannula				NIV	IMV
Etiology		Balf mNGS RSV-A						Blood culture *C. gilardii*	Sputum culture *C. gilardii*	
Lactic acid	2.7	-	2.4	3.7	3	5.2	3.9	1.6	1.5	4.7

WBC: White Blood Cell. PCT: Procalcitonin. PLT: Platelet. *C. gilardii*: *Cupriavidus gilardii*. RSV-A: Respiratory syncytial virus type A. NIV: Noninvasive ventilation. IMV: Invasive mechanical ventilation.

**Table 2:** Results of Blood and Sputum Bacterial Culture and Antimicrobial Susceptibility Testing.

**Blood Culture and Drug Sensitivity Results (*Cupriavidus*)**	
Drug	MIC value (ug/mL)
Ceftriaxone	2
Imipenem	≥32
Meropenem	≥32
Levofloxacin	0.125
**Sputum Culture and Drug Sensitivity Test Results (*Cupriavidus* 3+)**	
Drug	MIC value (ug/mL)
Ceftriaxone	1.5
Imipenem	>32
Meropenem	>32
Levofloxacin	0.094

**Table 3:** Case reports of *Cupriavidus* infection.

Reference	Age/Gender	Clinical Manifestations	Risk Factors	Etiological Sources	Treatment	Outcome
Daniel Tena et al. 2014 [3]	36 y/M	Fever, muscular abscess on the right thigh	History of renal transplantation, with the use of corticosteroids and immunosuppressive agents.	Pus culture shows *C. gilardii*	Surgical drainage and intravenous ciprofloxacin	Recovery
Xuejie Fang et al. 2023 [4]	78 y/M	Dyspnea, edema of limbs	COVID-19 pneumonia, history of corticosteroid use, prostate cancer post-surgery.	mNGS of balf and blood shows *C. gilardii*	Imipenem/cilastatin and trimethoprim-sulfamethoxazole	Death
Carmen Luna Aranaa et al. 2021 [5]	22 mo/M	Fever	ALL in reinduction phase, CVC carrier	Both blood cultures (peripheral and CVC) are *C. pauculus*	Maintained cefepime adding catheter lock therapy with ciprofloxacin; remove the catheter + intravenous cefepime after relapse.	Recovery
Tiziana D’Inzeo et al. 2015 [6]	70 y/M 62 y/M 26 y/F 73 y/M	All had fever	CHF rectal cancer placenta previa nasopharyngeal cancer (All had CVC carrier)	Both blood cultures (peripheral and CVC) are *C. metallidurans*	Catheter removal, piperacillin-tazobactam in two patients, the other two were treated with ciprofloxacin + meropenem	Recovery
Inês Gomes et al. 2021 [7]	90 y/M	Fever, disorientated and hypotensive	CKD, CHF, pacemaker implantation, MDS	blood cultures *C. pauculus*	Minocycline + Piperacillin/tazobactam	Death
Raghda Yahya et al. 2017 [8]	1 y/M	Dyspnea	Congenital medical, end-stage renal failure, recurrent aspiration pneumonia, tracheostomy	Sputum cultures *C. pauculus*	Cefepime	Death
Shan Tian et al. 2022 [9]	38 y/M	Fever, headache, sore throat, diarrhea	Graves hyperthyroidism with leucopenia induced	Bone marrow culture *C. pauculus*	Meropenem + glucocorticoid	Recovery
Stephanie H. Stovall et al. 2010 [10]	4 mo/M 3 y/M 1 mo/M 16 mo/F	Fever, vomiting, and lethargy - - -	Junctional ectopic tachycardia and refractory cardiogenic shock, requiring ECMO support Congenital heart defects, severe congestive heart failure, requiring ECMO support hypoplastic left heart syndrome, requiring ECMO support double outlet right ventricle, mitral stenosis, pulmonary stenosis, requiring ECMO support	blood cultures *C. pauculus* blood cultures *C. pauculus* blood cultures *C. pauculus* blood cultures *C. pauculus*	Meropenem + Gentamicin + piperacillin/tazobactam Cefepime + piperacillin/tazobactam Cefepime + ciprofloxacin Cefepime and 13 days of gentamicin	Recovery Recovery Death Recovery
Adaora S. Uzodi et al. 2014 [11]	15 mo/M	Fever	Hypoplastic left heart syndrome, heart transplant, requiring ECMO support	Both blood cultures (peripheral and CVC) are *C. pauculus*	Cefepime + ciprofloxacin	Recovery
Matthew Karafin et al. 2010 [12]	12 y/F	Fever, abdominal pain	Idiopathic aplastic anemia	Stool surveillance culture is *C. gilardii* Blood cultures *C. gilardii*	Cefepime + Amikacin Ciprofloxacin	Death
Sarela García-Masedo Fernández et al. 2019 [13]	27 y/M	Fever, abdominal pain, diarrhea, rectal bleeding	Ulcerative colitis	Blood cultures are *C. pauculus*	Ciprofloxacin	Recovery
Oh Joo Kweon et al. 2020 [14]	26 y/F	Fever	Acute myeloid leukemia, CVC carrier	Both blood (peripheral and CVC) mNGS are *C. gilardii*	Meropenem + teicoplanin	Death
Bianco G et al. 2018 [15]	48 y/F	Fever	acute myelomonocytic leukemia, CVC carrier	Both blood cultures (peripheral and CVC) are *C. pauculus*	Meropenem + Ciprofloxacin	Recovery
Emoke Almasy et al. 2016 [16]	67 y/M	Fever, respiratory distress	Epileptic, Alzheimer disease, chronic renal failure	Blood cultures are *C. pauculus*	Ciprofloxacin + ceftriaxone	Death
S Duggal et al. 2013 [17]	6 days/M	Fever, lethargy, Epileptic, icterus, facial grimace, occasional tonic posturing	environmental contamination during feeding	Cerebrospinal fluid and blood cultures are *C. pauculus*	Ceftazidime	Recovery
Banu AYDIN et al. 2012 [18]	16 days/M	Fever, cough and respiratory distress	Environmental pollution	Blood cultures are *C. pauculus*	Ceftazidime	Recovery
Ste’phanie Langevin et al. 2011 [19]	74 y/M	Fever	Colonic and bladder tumors, hypertension, and diabetes	Blood cultures are *C. metallidurans*	Piperacillin-tazobactam	Death
Syed A. Huda et al. 2020 [20]	41 y/F	Fever, flu-like symptoms, dyspnea	IVDU, infective endocarditis	Sputum culture is *C. pauculus*	Cefepime	Recovery
Zhen Zhang et al. 2017 [21]	87 y/M	Fever, dyspnea	COPD, hypertension	Blood cultures are *C. gilardii*	Piperacillin-tazobactam	Recovery
Takehito Kobayashi et al. 2016 [22]	90 y/F	Fever, wound suppuration	Pacemaker implanted	Secretion and blood culture are *C. gilardii*	Cut the lead, ampicillin-sulbactam + cefepime + Ciprofloxacin	Recovery
Joshua B. Christensen et al. 2010 [23]	29 y/F	Wound pain and swelling	ICD placed	Blood cultures are *C. pauculus*	Levofloxacin	Recovery
Mahesh B. Shenai et al. 2019 [24]	58 y/F	Wound suppuration	Refractory Parkinson’s disease, postoperative period of DBS	The connecting lead culture are *C. pauculus*	The generator and the connecting lead were explanted, ceftazidime	Recovery
Kalka-Moll, W.M. et al.2009 [25]	11 y/M 22 y/M	Expectorate, dyspnea Symptoms of a mild upper respiratory tract infection	CF, trisomy 21, operative correction of a patent ductus arteriosus CF, congenital myotonia	Throat swab cultures is *C. pauculus* Sputum culture is *C. respiraculi*	Amoxicillin-clavulanate Untreated	Recovery Recovery
Liu, Y. Gao, J. et al.2025 [26]	87 y/M	Fever	Acute myocardial infarction and CHF	Sputum culture showed growth of *C. gilardii*	Cefoperazone sulbactam + minocycline	Death

M = male, F = female. Age in years (y), months (mo) or days. ALL = acute lymphoblastic leukemia; CVC = central venous catheter; CKD = chronic kidney disease; CHF = congestive heart failure; MDS = myelodysplastic syndrome; ECMO = extracorporeal membrane oxygenation; IVDU = intravenous drug use; COPD = chronic obstructive pulmonary disease; ICD = implantable cardiac defibrillator; DBS = deep brain stimulation; CF = cystic fibrosis.

**Table 4:** Summary of *Cupriavidus* infection cases.

Variables	Median (IQR) or N (%)
Age	32.5 (2.12–70) (Range: From 6 days after birth to 90 years old)
Sex (M/F)	23/9 cases
Risk factors or comorbidity	
Non-immunosuppressive state	1 (3.1%)
Environmental factors	2 (6.3%)
Solid organ transplantation	2 (6.3%)
Hematological diseases	6 (18.8%)
Use of glucocorticoids	3 (9.4%)
Use of immunosuppressive agents	2 (6.3%)
History of preceding infection	3 (9.4%)
Cardiovascular disease	11 (34.4%)
Diabetes	2 (6.3%)
Renal insufficiency	3 (9.4%)
Malignant tumors (nonhematologic)	4 (12.5%)
Autoimmune diseases	2 (6.3%)
Congenital diseases	7 (21.9%)
Intravascular invasive procedures	18 (56.3%)
Intratracheal invasive procedures	2 (6.3%)
Clinical manifestations	
Fever	23 (71.9%)
Respiratory symptoms	10 (31.3%)
Gastrointestinal symptoms	5 (15.6%)
Neurological symptoms	4 (12.5%)
Skin and soft tissue infections	5 (15.6%)
Site of infection	
Bloodstream and Catheter-related Infections	24 (75%)
Respiratory tract	7 (21.9%)
Gastrointestinal	1 (3.1%)
Skin and soft tissue	4 (12.5%)
Central nervous system	1 (3.1%)
Bone marrow	1 (3.1%)
Strain Classification	
*C.* *pauculus*	18 (56.3%)
*C.* *gilardii*	8 (25%)
*C.* *metallidurans*	5 (15.6%)
*C.* *respiraculi*	1 (3.1%)
Clinical Outcome	
Recovery	22 (68.7%)
Death	10 (31.3%)

## Data Availability

The data and materials can be obtained by contacting the corresponding author via email.
